# Polyethylene Glycol-Based Solid Polymer Electrolyte with Disordered Structure Design for All-Solid-State Lithium-Ion Batteries

**DOI:** 10.3390/mi16101123

**Published:** 2025-09-30

**Authors:** Wanlin Wu, Yingmeng Zhang, Zhongke Zhao, Yihan Lin, Yongliang Li, Xiangzhong Ren, Peixin Zhang, Lingna Sun

**Affiliations:** 1 College of Chemistry and Environmental Engineering, Shenzhen University, Shenzhen 518060, China; 2 Yangtze Delta Region Institute (Huzhou), University of Electronic Science and Technology of China, Huzhou 313000, China

**Keywords:** polyethylene glycol, disordered structure, Lewis acid groups, anion capture ability, polymer electrolyte

## Abstract

In this work, a novel solid polymer electrolyte with a disordered structure has been designed, combining polyethylene glycol (PEG) as the flexible segments and hexamethylene diisocyanate (HDI) as the rigid segments. The synthesis was realized by alternating flexible PEG with rigid HDI through a peptide bond (–CO–NH–), which disrupts the ordered structures of PEG, generating electron-deficient Lewis acid groups. The pathbreaking introduction of HDI blocks not only bridges links between the PEG molecules but also generates electron-deficient Lewis acid groups. Therefore, the original ordered structures of PEG are disrupted by both the alternating chains between PEG and HDI and the Lewis acid groups. As a result, the PEG_H/L4000_ electrolytes (PEG molecular weight of 4000) exhibit a strong anion-capture ability that decreases the crystallinity of polymers, which further achieves a high ionic conductivity close to 10^−3^ S·cm^−1^ with the lithium-ion transference numbers up to 0.88. The symmetric Li|PEG_H/L4000_|Li cells maintain a low and stable voltage polarization for more than 800 h at 0.1 mA·cm^−2^. Furthermore, the LiFePO_4_|PEG_H/L4000_|Li all-solid-state cells perform well both in cycling and rate performances. The design of polymer disordered structures for polymer electrolytes provides a new thought for manufacturing all-solid-state lithium-ion batteries with high safety as well as long life.

## 1. Introduction

With a developing economy, people have higher requirements for energy, and the quantity demanded has increased day by day [1]. Lithium-ion batteries (LIBs) have become a promising choice, which combine the advantages of clean energy, high energy density, and long lifespan performance [2]. However, commercial LIBs with liquid electrolytes have obvious shortcomings of potential safety risks and limited mechanical properties [3,4]. Therefore, solid electrolytes come into consideration, which can be divided into inorganic solid electrolytes and polymer solid electrolytes. Due to the high interface impedance and poor interfacial stability of inorganic solid electrolytes, polymer solid electrolytes have become better choices [5].

The discovery of conductivity in polyethylene oxide (PEO) by Wright in 1973 [6] is regarded as the beginning of the development of polymer electrolytes. After that, PEO-based electrolytes quickly became a research hotspot. With the in-depth exploration of research works, Berthier noted that the low ionic conductivity of PEO-based electrolytes (<10^−7^ S·cm^−1^) was principally caused by the high crystallinity [7]. These discoveries have inspired various modification research on polymer electrolytes.

Modifications of PEO-based electrolytes can be categorized into macro- and micro-scale methods. Macroscopic modifications include introducing various additives [8,9,10], building multi-layered structures [11,12,13] or nano-scaffold frameworks [14,15,16], and covering artificial solid electrolyte interphase (SEI) layers [17,18,19]. The capacity and cycling performance of LIBs can be specifically improved by the macroscopic modifications; however, the impedance of the electrolytes has been increased to a large extent, which has a negative effect on the electrochemical performances and fails to meet the requirements for high-performance batteries. As for comparisons, the microscopic modifications focus on the molecular structure engineering of PEO to avoid the above shortcomings. Some scholars are inserting structural units into PEO molecular chains by disordered or block copolymerization, or grafting branched chains with thermodynamic and chemical/electrochemical stability onto PEO chains [20,21,22,23]. The structural units can successfully moderate the molecular structure and prevent the crystallization of PEO [20,21,22,23]. Furthermore, the inserted structural units are usually hard segments, while PEO is a soft segment; the polymers with hard and soft segments alternated can change the conductivity and mechanical properties of the electrolyte simultaneously [24].

In addition, some scholars synthesized three-dimensional hyperbranched polymers [17,25] with a number of branched chains and chain ends; such polymers are in an almost amorphous state, which is beneficial for transporting lithium ions. However, with the increase in side chains, the mechanical stability of the polymer electrolytes is reduced, and the electrolytes might even appear as viscous liquids. In order to change this phenomenon, scholars designed the molecular structure of polymer electrolytes with chemical cross-linkages between polymer chains [26,27,28], which means the small-weight molecules as side chains are replaced by large-weight molecules for mechanical stability. As a result, molecular structure engineering can successfully increase the dissociation of lithium salts by reducing the crystallinity and glass transformation temperature (Tg) of the main chains or side chains of PEO, which finally improves the conductivity of polymer electrolytes.

However, as ether-type oxygen atoms in PEO are kinds of Lewis alkalis, Lewis acid–base reactions that have happened with lithium ions are much stronger than the reactions with anions. The traditional polymer solid electrolyte modifications mentioned above ignore this vital point; thus, the traditional PEO-based electrolytes are bound to sacrifice part of the lithium-ion (Li^+^) transferability as a premise to achieve high conductivity. Therefore, polymer electrolytes with high permittivity and many electron-deficient Lewis acid groups have become better choices. The introduction of electron-deficient Lewis acid groups into polymer electrolytes can achieve the dual purpose of improving conductivity and lithium-ion transference numbers, which can capture anions so as to increase the number of free lithium ions [29]. As an efficient Li^+^ conductive polymer, polyethylene glycol (PEG) can effectively expedite Li^+^ diffusion but hinder anion migration that allows Li^+^ to migrate through interconnected interfaces, which provides polymer electrolytes with an enhanced diffusion coefficient and high Li^+^ transference number [30,31]. Compared to PEO with long spiral chain structures, PEG with low molecular weights has high internal rotation energy barriers, in which lithium ions are prone to trans-coordination and are beneficial to the transmission [32,33].

In this work, polymer electrolytes PEG_H/L4000_ were synthesized by disrupting the ordered structure of PEG with a 4000 molecular weight, which combined rigid and flexible chains without compatibility problems. Moreover, Lewis acid groups (–C=O and –COO^−^) were successfully introduced on the chains of PEG_H/L4000_. Furthermore, the lithium ions were grafted onto the rigid segments of PEG_H/L4000_ to ensure the mobility of the conducting ions in the hard phase. The PEG_H/L4000_ electrolyte shows a high ionic conductivity close to 10^−3^ S·cm^−1^ with Li^+^ transference numbers up to 0.88 and an electrochemical stability window of 5.18 V (vs. Li^+^/Li). The Li|PEG_H/L4000_|Li cells maintain a low and stable polarization for more than 800 h at 0.1 mA·cm^−2^. The LiFePO_4_|PEG_H/L4000_|Li cells show a high initial coulombic efficiency of 96.5% and an initial specific capacity of 163.1 mAh·g^−1^, performing well in both cycling and rate performances while maintaining a coulombic efficiency of 99.6% in the 200th cycle at 0.1 C.

## 2. Materials and Methods

Material: Polyethylene glycol (PEG, Mw = 2000 g mol^−1^), polyethylene glycol (PEG, Mw = 4000 g mol^−1^), 2,2-dimethylolpropionic acid (DMPA, 98.0%), lithium hydroxide (LiOH, 98.0%), lithium bis(trifluoromethane sulfonimide) (LiTFSI, 99.0%) and N-methyl pyrrolidone (NMP, >99.5%) were from Macklin, Shanghai, China. Hexamethylene diisocyanate (HDI, 99.0%) and N,N-dimethylformamide (DMF, >99.8%) were from Aladdin, Pico Rivera, CA, USA. Dibutyltin dilaurate (DBTL, 90.0%) was from Meryer, Vallejo, CA, USA. Ferrous lithium phosphate (LiFePO_4_), poly(1,1-difluoroethylene) (PVDF), and acetylene blacks were from Kejing Star Technology, Shenzhen, China. Separators (Celgard 2325 PP/PE/PP separator) were from Celgard, Charlotte, NC, USA. Polyethylene glycol (PEG, Mw = 2000 g mol^−1^) and polyethylene glycol (PEG, Mw = 4000 g mol^−1^) needed to be placed in a vacuum oven at 80 °C for 24 h to remove the water before the experiment. Molecular sieves (4A) were used to remove the water contained in the DMF. LiTFSI, DMPA, and LiOH needed to be stored in an argon-filled glove box (O_2_ < 0.1 ppm, H_2_O < 0.1 ppm).

Synthesis of LiDMPA: Lithium 2,2-dimethylolpropionate (LiDMPA) was prepared by adding 0.01 mol of 2,2-dimethylolpropionic acid (DMPA) and 0.01 mol of lithium hydroxide (LiOH) together with 10 mL of distilled water in a round-bottom flask in a 60 °C oil bath with mechanical stirring for 4 h. When the reactions were completed, the solution needed to be placed in a vacuum oven at 120 °C for 24 h to remove water from the white crystal products and stored in an argon-filled glove box (O_2_ < 0.1 ppm, H_2_O < 0.1 ppm).

Synthesis of PEG_H_: 40 mL N,N-dimethylformamide (DMF), 0.004 mol polyethylene glycol (PEG) with different molecular weights, 0.008 mol hexamethylene diisocyanate (HDI), and 20 μL dibutyltin dilaurate (DBTL) were added into a three-mouth flask in an argon atmosphere in an 80 °C oil bath with mechanical stirring for 2 h, which is named PEG_H_ as an intermediate product.

Synthesis of PEG_H/L_: 0.0045 mol LiDMPA was added into PEG_H_ in an argon atmosphere in an 80 °C oil bath and mechanically stirred for 2 h. Lithium bis-trifluoromethanesulfonimide (LiTFSI), with the molar ratio of ethylene oxide units to lithium ions (EO:Li) approximately equal to 18, was added to the above mixture under the same conditions for 6 h. Then, the solution was uniformly coated on a Teflon model and dried in a vacuum oven at 60 °C for 48 h to remove the DMF solvent. The dried membranes were stored in an argon-filled glove box (O_2_ < 0.1 ppm, H_2_O < 0.1 ppm) and marked as PEG_H/L2000_ and PEG_H/L4000_.

Synthesis of PEG_LiTFSI_: As a comparison sample, PEG_LiTFSI_ was synthesized by mixing PEG directly with LiTFSI, with an EO:Li equal to 18, in an argon atmosphere in an 80 °C oil bath and mechanically stirred for 6 h, coated on a Teflon model, and dried in a vacuum oven at 60 °C for 48 h.

Synthesis of cathodes: Ferrous lithium phosphate (LiFePO_4_) and acetylene blacks with poly(1,1-difluoroethylene) (PVDF) were mixed at a weight ratio of 8:1:1, and N-methyl pyrrolidone (NMP) was used as the solvent. The mixture was stirred at 300 rpm for 24 h and coated on aluminum foils evenly. The aluminum foils were dried in an oven at 60 °C for 12 h to remove the NMP solvent.

Material characterization: To confirm the successful synthesis of PEG_H/L_, the mid and final materials obtained at each step were characterized by Fourier transform spectroscopy (FTIR). A total of 10 mg of PEG_H/L_ with different molecular weights was dissolved in 500 μL dimethyl sulfoxide (DMSO) and tested by a VNMRS-400 nuclear magnetic resonance (NMR) spectrometer to obtain the NMR spectra. X-ray diffraction (XRD) of PEG_LiTFSI_ and PEG_H/L_ was obtained by Cu K radiation in an empyrean diffractometer at scanning rates ranging from 10° to 90° at a scan rate of 0.05°/s. The morphology was analyzed by field-emission scanning electron microscopy (FESEM, JSM-7800F & TEAM Octane Plus, 10 kV, Tokyo, Japan). A differential scanning calorimeter (DSC, DSC-200F3) was used to characterize the glass transition temperature (T_g_) of the polymers, with an experimental temperature range from −100 °C to 0 °C at a heating rate of 10 K/min. Thermogravimetric analysis (TGA, STA409PC) was performed on PEG_H/L_ in a nitrogen atmosphere with a temperature range from 50 °C to 1000 °C at a heating rate of 10 K/min.

Density functional theory (DFT) calculation in Binding energies: All the spin-polarized calculations based on density functional theory (DFT) were performed utilizing the DMol3 package [34]. The generalized gradient approximation (GGA) in the Perdew–Burke–Ernzerhof form and Semicore Pseudopotential method (DSPP) with the double numerical basis sets plus the polarization functional (DNP) were adopted [35,36]. A (3 × 3 × 1) supercell was used. The Brillouin zones were sampled using the Monkhorst–Pack scheme with a 3 × 3 × 3 k-point grid, and the double numerical basis sets plus the polarization functional (DNP) were adopted. A DFT-D correction with the Grimme scheme was used to account for the dispersion interaction [37]. The SCF convergence for each electronic energy was set as 1.0 × 10^−5^ Ha, and the geometry optimization convergence criteria were set up as follows: 1.0 × 10^−5^ Ha for energy, 0.004 Ha Å-1 for force, and 0.01 Å for displacement. Energy barriers were examined by linear and quadratic synchronous transit methods in combination with the conjugated gradient (CG) refinement. Finally, the binding energies (BEs) were calculated as BE= Ead/sub -Ead -Esub, where Ead/sub, Ead, and Esub are the total energies of the optimized adsorbate/substrate system, the adsorbate in the structure, and the clean substrate, respectively.

Electrochemical measurements: The PEG_LiTFSI_ and PEG_H/L_ with different molecular weights were assembled into Li|PEG_LiTFSI_|Li cells and Li|PEG_H/L_|Li cells. The lithium-ion transference numbers (t_Li+_) were tested by the methods of chronoamperometry (CA) to record the current change within 1000 s and A.C. impedance spectroscopy for the impedance before and after the CA test. Lithium-ion transference numbers were calculated using Equation (1):

(1) $$t_{Li+}=\frac{I_{\mathrm{SS}}\times \left(\Delta V-I_{0}R_{0}\right)}{I_{0}\times \left(\Delta V-I_{\mathrm{SS}}R_{\mathrm{SS}}\right)}$$

where I_SS_ and I_0_ are respectively referred to as the steady and initial current, R_0_ and R_SS_ are the initial and steady block resistance, respectively, and ΔV is the polarization voltage.

The assembled PEG_LiTFSI_, PEG_H/L2000_, and PEG_H/L4000_ were placed into a symmetric cell configuration of Stainless steel (SS)|Electrolyte|Stainless steel (SS), denoted as SS|PEG|SS. The use of electrochemically inert SS electrodes ensures that any measured current is solely due to electronic conduction through the electrolyte, as no Faradaic reactions (e.g., lithium plating/stripping or corrosion) can occur within the applied voltage window. The impedance was tested by electrochemical impedance spectroscopy (EIS) with an AC amplitude of 10 mV in the frequency range of 10^6^ to 0.01 Hz. The electronic conductivity of PEG_H/L_ at room temperature was tested by a direct current (DC) polarization method arranged from −2 V to 2 V on a symmetric cell of SS|PEG|SS, where the electrolyte membrane was sandwiched between two blocking stainless steel electrodes. The electronic conductivity was calculated using Equation (2):

(2) $$\sigma =\frac{L}{R\times S}$$

where σ (S·cm^−1^) is referred to as the conductivity, L (cm) is the thickness of the electrolyte films, S (cm^2^) is the area of the electrolyte films, and R (Ω) is the resistance of the stainless steel cells (SS|PEG|SS cells) tested by EIS.

The assembled PEG_LiTFSI_, PEG_H/L2000_, and PEG_H/L4000_ were placed into the SS|PEG|Li cells, and the electrochemical windows were tested by linear sweep voltammetry (LSV) in the range of 0 V to 6 V. Cyclic voltammetry (CV) was performed with Li | PEG_H/L_ | LiFePO_4_ cells at 60 °C. All tests above were conducted in the Solartron electrochemical station 1260 A + 1470 E. Rate performances and cycling performances of PEG_H/L2000_ and PEG_H/L4000_ were tested at 60 °C with the voltage range from 2.5 V to 4.5 V in LiFePO_4_|PEG_H/L_|Li all-solid-state cells in LAND CT2001A.

## 3. Results and Discussion

**Figure 1:**
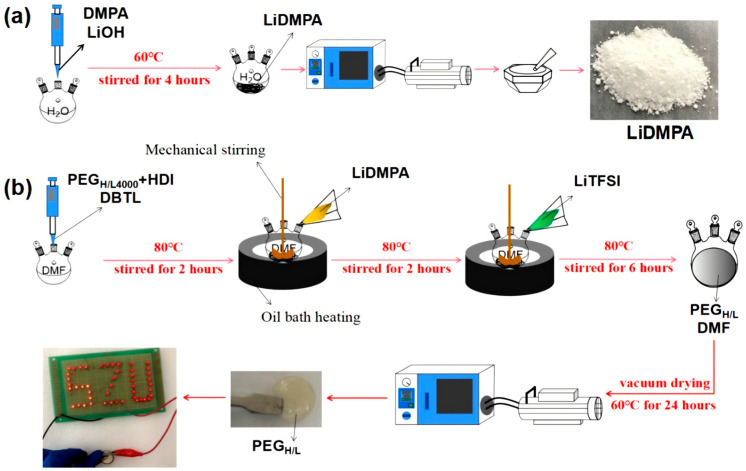
Schematic illustration of the preparation processes of solid electrolyte PEG_H/L_ with a disordered structure: (**a**) preparation process of LiDMPA; (**b**) preparation process of PEG_H/L_.

The synthesis procedures of PEG_H/L_ are detailed in Figure 1 and Figure S1a. In the first step, lithium 2,2-dimethylolpropionic acid (LiDMPA) was prepared as the precursor with the purpose of grafting lithium ions in the hard segments of the solid electrolytes (Figure 1a), which was synthesized through an acid–base neutralization reaction between lithium hydroxide (LiOH) and 2,2-dimethylolpropionic acid (DMPA) (Figure S1(aI)). The second step is to realize the alternations of hard and soft segments for the intermediate products, named PEG_H_ (Figure S1(aII)), by generating carbamates (–CO–NH–) through the reaction between –N=C=O groups in hexamethylene diisocyanate (HDI) and –OH groups in polyethylene glycol (PEG) with different molecular weights, in which the HDI acts as the hard segments and PEG acts as the soft segments (Figure 1b).

The molar ratio of HDI to PEG is a critical parameter as it determines the cross-linking density and the concentration of Lewis acid groups (–C=O). In this research design, an optimal HDI/PEG ratio was fixed at 2:1 based on stoichiometric calculations for the complete reaction, ensuring all PEG chains were effectively linked and leaving sufficient unreacted –NCO groups from HDI to subsequently react with LiDMPA (Figure S1(aII)). A higher HDI ratio could lead to excessive cross-linking, reducing chain mobility and thus ionic conductivity, while a lower ratio would result in insufficient formation of the disordered structure and Lewis acid sites, failing to effectively disrupt crystallinity and capture anions.

In the third step, a block copolymer was obtained with the characteristic functional group reactions occurring between the –N=C=O groups in PEG_H_ and –OH groups in LiDMPA to generate carbamates (Figure S1(aIII)), in which the –N=C=O groups from HDI have not been completely consumed in the previous reaction (Figure S1(aII)) and can be further reacted with the –OH groups in this part, where –OH groups were grafted on the LiDMPA in the first step (Figure S1(aI)). As a result, the grafted lithium ions on the LiDMPA were successfully linked to the block copolymer PEG_H_ and helped to realize the transfer of lithium ions in the hard segments of the solid electrolytes (Figure S1(aIII)). Finally, final products named PEG_H/L_ (Figure S1(aIV)) are obtained by mixing the above block copolymer products (in Figure S1(aIII)) with lithium bis(trifluoromethane sulfonimide) LiTFSI salts, according to the molecular weight of PEG; they are named PEG_H/L2000_ and PEG_H/L4000_, respectively.

The novel PEG_H/L_ solid electrolytes improve the conductivity of polymer electrolytes [38,39]. Although the existence of rigid segments will cause microphase separation, the whole system has successfully solved the compatibility problem by distributing discontinuous rigid segments (HDI) in flexible segments (PEG) [40]. HDI is introduced to disrupt the ordered structure of PEG by blocking soft segments; therefore, the crystallinity of PEG is reduced, and the lithium-ion transference capability in the electrolyte has been improved. At the same time, Lewis acid groups (–COO^−^ and –C=O) are also generated during the reactions between HDI and PEG. When the anions move near to PEG_H/L_, strong Lewis acid–base reactions occur simultaneously between the anions as Lewis alkali groups and the –C=O and –COO^−^ as Lewis acid groups, which correspond to two different reaction mechanisms. When anions move near to –C=O, σ bonds are formed by electron pairs and carbonyl groups [41,42]. According to the octet rule [43], electron pairs from the carbon–oxygen double bonds are closer to the more electronegative oxygen atoms, that is to say, the anions are more likely to bond with the oxygen in –C=O for Lewis acid–base pairs. When anions move near to –COO^−^, anions are captured by –COO^−^ in Lewis acid–base reactions to form the intermediates, accompanied by electron pair transfer. As a result, the lithium-ion transference of the solid electrolytes has been successfully improved with the above two modifications by introducing HDI into PEG molecular chains and capturing anions through a Lewis acid–base neutralization reaction to release free Li^+^ ions.

In another experiment (Figure S1b), PEG and LiTFSI were directly mixed to prepare the comparison sample, named PEG_LiTFSI_. PEG_LiTFSI_ can be seen as the traditional PEG-based solid electrolyte. In traditional PEG-based PEG_LiTFSI_, PEG molecules with high molecular weight are entangled with each other, hindering the transference of lithium ions (Figure S1(cI)). Through the characteristic functional group reaction between the soft segment PEG and the hard segment HDI, the PEG molecules can be end-to-end connected by –N=C=O groups from the HDI blocks and distributed neatly in the solid electrolytes (Figure S1(cII)). Furthermore, in order to take full advantage of the hard segments, lithium ions are also grafted onto the HDI rigid chains for realizing the transference of lithium ions in the hard segments (Figure S1(cIII)). It should be pointed out that the hard segment HDI blocks are the most important key for preparing the novel PEG_H/L_ solid electrolytes, which not only perform like bridges for linking the PEG molecules but also provide sites for transforming into the electron-deficient Lewis acid groups (–C=O) and indirectly grafting lithium ions from LiDMPA.

In addition, the precursor materials (PEG, DMPA, HDI) and the synthesis solvent (DMF) are water-soluble or miscible. The final cross-linked PEG_H/L_ polymer network, however, is insoluble in water, which is a critical property for a stable solid electrolyte in a battery. Therefore, the use of water-soluble precursors is advantageous for environmentally friendlier processing routes compared to some other polymer synthesis methods. While the cross-linked final product is designed to be stable and inert, some precursor materials, like HDI, require careful handling. It encapsulates these monomers into a stable, non-volatile matrix, mitigating potential handling hazards in the final application compared to liquid electrolytes.

**Figure 2:**
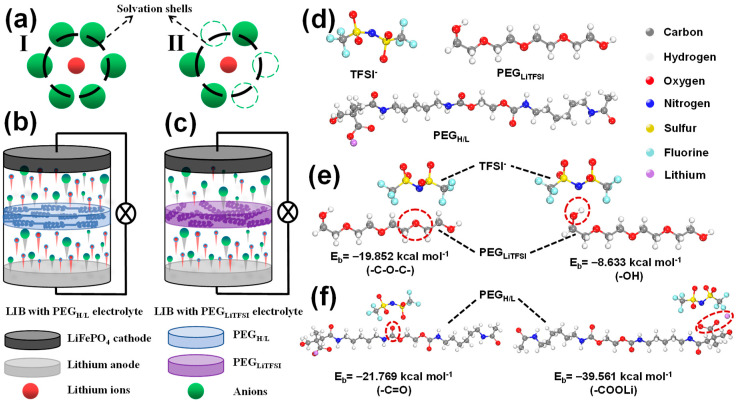
(**a**) Lithium-ion solvation models in PEG_LiTFSI_ and PEG_H/L_ molecules; schematic illustrations of the lithium-ion distribution in LIBs with (**b**) PEG_LiTFSI_ and (**c**) PEG_H/L_; DFT calculation models of (**d**) PEG_LiTFSI_, PEG_H/L_, and LiTFSI, as well as (**e**) PEG_LiTFSI_ and (**f**) PEG_H/L_ binding with TFSI^−^.

To better understand the working mechanism of the novel PEG_H/L_ solid electrolytes, a solvation model has been introduced to explain the behavior (Figure 2a). In a traditional PEG-based PEG_LiTFSI_ electrolyte prepared by directly mixing PEG polymers with LiTFSI salts, lithium ions are usually surrounded by 5–6 TFSI^−^ anions. This phenomenon is called the solvation of lithium ions (Figure 2(aI)). However, in the PEG_H/L_ electrolytes, the strong Lewis acid–base reaction occurs between TFSI^−^ anions and –C=O/COO^−^ Lewis acid groups in PEG_H/L_, which reduces the number of TFSI^−^ anions surrounding lithium ions and relieves the solvated lithium ions (Figure 2(aII)). Figure 2b and Figure 2c, respectively, illustrate the lithium-ion distribution in the LIBs during the charge–discharge processes. As shown in Figure 2b, because of the strong Lewis acid–base reactions between TFSI^−^ and PEG_H/L_, a large number of lithium ions are released with weakened solvation. The significantly reduced solvation of lithium ions in PEG_H/L_ mitigates the build-up of a severe concentration gradient compared to systems with traditional PEG-based PEG_LiTFSI_ electrolytes. By contrast, in Figure 2c, the strong solvation of lithium ions in the PEG_LiTFSI_ electrolytes impedes the transference of lithium ions. Some lithium ions fail to pass through the PEG_LiTFSI_ electrolytes, which further brings about a serious concentration polarization phenomenon.

To further testify to the introduction of Lewis acid groups, which can make contributions to capturing anions from LiTFSI for releasing more free lithium ions, the calculations based on density functional theory (DFT) have been established, as shown in Figure 2d–f. Figure 2d reveals the PEG_LiTFSI_, PEG_H/L_, and TFSI^−^ models, and the binding energies (BEs) of –C–O–C–, –OH, –C=O, and –COO^−^ interacting with TFSI^−^ were calculated based on the DFT method, as exhibited in Figures S1b and S2c. The BE with TFSI^−^ of –C–O–C– and –OH for PEG_LiTFSI_ (Figure 2e) are calculated to be −19.852 kcal·mol^−1^ and −8.633 kcal·mol^−1^, while BE with TFSI^−^ of –C=O and –COO^−^ for PEG_H/L_ (Figure 2f) are calculated to be −21.769 kcal·mol^−1^ and −39.561 kcal·mol^−1^, respectively, which prove that the BE of –C=O and –COO^−^ with TFSI^−^ are stronger than that of –C–O–C– and –OH. The DFT calculation is to quantitatively show that the functional groups in the designed PEG_H/L_ polymer have a much stronger affinity for anions than those in traditional PEG_LiTFSI_. Therefore, it is attested that the introduction of Lewis acid groups, –COO^−^ and –C=O, can successfully enhance the ability of PEG_H/L_ to capture anions from lithium salts, providing a mechanistic basis for the observed high transference number shown below.

**Figure 3:**
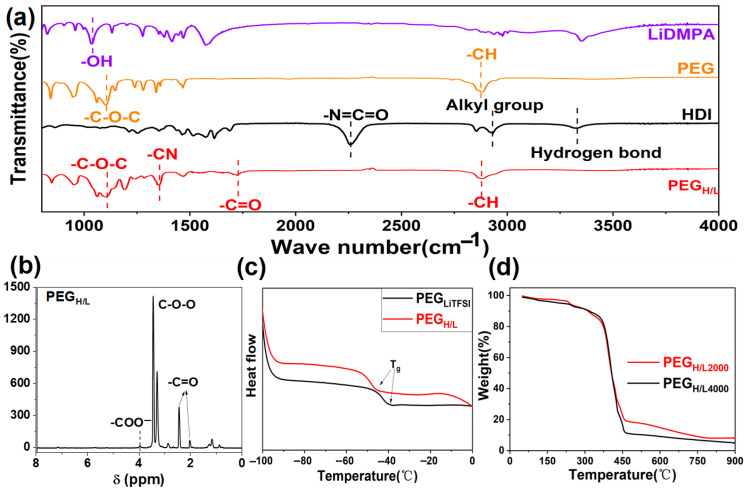
(**a**) FTIR spectra of LiDMPA, PEG, HDI, PEG_H/L_; (**b**) ^1^H-NMR spectrum of PEG_H/L_; (**c**) DSC curves of PEG_LiTFSI_ and PEG_H/L_; (**d**) TGA curves of PEG_H/L_.

To confirm the successful synthesis of PEG_H/L_, the materials obtained at each synthesis step were characterized by Fourier transform spectroscopy (FTIR). The spectra in Figure S2 show the absorption peaks of 2,2-dimethylolpropionic acid (DMPA) and lithium 2,2-dimethylolpropionic acid (LiDMPA). The absorption peak at 1680 cm^−1^ is assigned to the –COOH from DMPA and turns into two new absorption peaks from –COO^−^ in LiDMPA after the reactions between lithium hydroxide (LiOH) and DMPA, which are located at 1417 cm^−1^ and 1593 cm^−1^, respectively [44]. It proves that the reactions between LiOH and DMPA were successfully achieved, where the Li^+^ replaced the H^+^ in the –COOH groups of DMPA to form –COOLi groups. The absorption peaks of LiDMPA, HDI, PEG, and PEG_H/L_ are shown in Figure 3a. The absorption peaks at 1106 cm^−1^ and 2882 cm^−1^ are, respectively, assigned to the –C–O–C– and –CH from PEG. The absorption peak at 2325 cm^−1^ is ascribed to –N=C=O in HDI. The spectrum of PEG_H/L_ shows the new absorption peaks at 1352 cm^−1^ and 1535 cm^−1^, which, respectively, refer to –CN and –C=O. By comparing the spectra of LiDMPA, PEG, and HDI with PEG_H/L_, the complete disappearance of the –N=C=O peak from HDI and the –OH peak from LiDMPA in the final PEG_H/L_ FTIR spectrum, coupled with the appearance of new carbamate (–CN and –C=O) peaks, confirms the consumption of HDI monomers and their covalent bonding to both PEG and LiDMPA. This confirms the incorporation of HDI into the polymer chain, which means the reactions described in Figure S1a take place and the target product PEG_H/L_ has been successfully synthesized.

According to the nuclear magnetic resonance (NMR) characterizations in Figure 3b, the existence of –COO^−^ and –C=O peaks also proves the successful synthesis of PEG_H/L_ [45], which is different from the ^1^H-NMR spectra of DMPA, LiDMPA, and HDI reactants (Figure S3). The microscopic morphology of the PEG_H/L_ membranes was investigated by a field emission scanning electron microscope (FESEM), as shown in Figure S4. The smooth surface is beneficial to form a good interface with the electrodes. Comparing the X-ray diffraction (XRD) patterns in Figure S5, PEG_H/L_ shows less crystallinity than PEG_LiTFSI_, which contributes to a higher ionic conductivity. Because the introduction of HDI and LiDMPA increases the amorphous phase ratio in the polymers, which is beneficial to improve the conductivity and the transferability of lithium ions, the free diffusion of lithium ions is mainly contributed by the amorphous phase of the polymers [46,47]. The differential scanning calorimeter (DSC) test of the electrolytes from Figure 3c exhibits the glass transition temperature (Tg) of PEG_H/L_, which is lower than PEG_LiTFSI_ and further indicates the higher amorphous phase proportion of PEG_H/L_. Figure 3d shows the thermogravimetric analysis (TGA) of PEG_H/L2000_ and PEG_H/L4000_, which attests to their good thermal stability with no obvious thermogravimetric weight loss before 370 °C. The significant reduction in crystallinity (XRD, Figure S5) and the lower Tg (DSC, Figure 3c) of PEG_H/L_ compared to PEG_LiTFSI_ provide strong indirect evidence that the HDI segments have successfully disrupted the ordered structure of PEG, supporting a model where rigid HDI segments are distributed within the flexible PEG matrix. Primarily, the disruption of long-range crystallinity by inherent disorder promotes highly amorphous phases, thereby enabling enhanced segmental motion and ultimately yielding superior ionic conductivity, particularly at ambient temperatures [38,39].

To evaluate the performance of solid electrolytes, it is crucial to quantify the mechanical properties, particularly for suppressing lithium dendrite growth. Significant efforts have been made to conduct standard tensile tests on the PEG_H/L_ and PEG_LiTFSI_ films. However, a considerable practical challenge has been encountered due to the intrinsic material properties of the synthesized polymer electrolytes. The films exhibit a pronounced tackiness and strong adhesive nature, which are inherent to the highly flexible PEG chains and the designed molecular structure. During tensile testing, this high adhesion caused the PEG_H/L_ samples to prematurely detach at the grips rather than in the gauge section, making it extremely difficult to obtain reliable and reproducible stress–strain data. This is a common issue when testing soft, highly adhesive polymer films. Similarly, the pure PEG_LiTFSI_ membrane was much softer and more sticky, demonstrating inferior mechanical integrity, which made it difficult to handle for testing.

The fundamental improvement in mechanical strength can be theoretically explained by the molecular design of the electrolyte:

*Role of rigid segments (HDI)*: The incorporated HDI blocks act as reinforcing points within the soft PEG matrix. These rigid segments restrict the segmental motion of the polymer chains, significantly enhancing the modulus and tensile strength of the overall material [27].

*Synergy of hard and soft segments*: This design creates a rigid–flexible coupled system. The flexible PEG segments facilitate ion transport, while the rigid HDI segments provide the mechanical scaffold, successfully decoupling and simultaneously improving both ionic conductivity and mechanical strength [24,30].

*Formation of a cross-linked network*: The chemical reaction between the flexible PEG chains (soft segment) and the rigid HDI blocks (hard segment) creates a covalently cross-linked polymer network, effectively distributing mechanical stress throughout the material [26,28].

**Figure 4:**
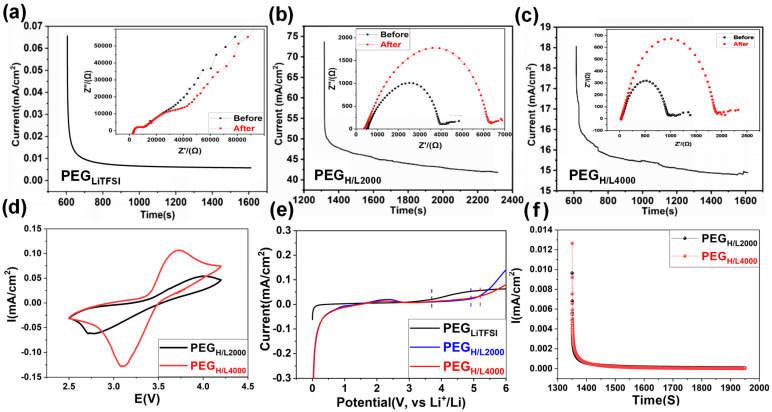
Lithium-ion transference number tests for (**a**) PEG_LiTFSI_, (**b**) PEG_H/L2000,_ and (**c**) PEG_H/L4000_, showing chronoamperometry curves (main) and corresponding electrochemical impedance spectra before and after polarization (insets). (**d**) Cyclic voltammetry of electrolytes at 60 °C, (**e**) electrochemical windows range from 0 V to 6 V, (**f**) electronic conductivity of electrolytes at room temperature.

For the traditional PEG-based electrolytes, PEG_LiTFSI_ is prepared by directly mixing PEG polymers with LiTFSI salts. On account of the lack of electron-deficient structures, there are almost no reactions between PEG and TFSI^−^ anions; therefore, anions act as free particles that incur serious self-discharge and surface degradation of electrolytes for LIBs [48]. More seriously, strong coordination effects between ether oxygen atoms and lithium ions are caused by the strong electron-providing ability, resulting in crosslinking between polyether molecules and giving rise to high microscopic viscosity of polymers. Furthermore, the weak movement ability of chains and transferability of lithium ions in polymer electrolytes generate low lithium-ion transference numbers (t_Li+_), which are usually less than 0.5. The t_Li+_ is calculated from Figure 4a–c according to Equation (1), which is tested by the chronoamperometry (CA) method, as well as the A.C. impedance spectroscopy testing for the impedance before and after the CA test (Figure 4a–c, inserts).

The t_Li+_ of PEG_LiTFSI_ is calculated to be a very low value of 0.06 (Figure 4a). Generally, the low t_Li+_ signifies a high anion transference number (t_−_ = 1 − t_Li+_), which stands for strong anion transferability. As the transfer pathways of anions are opposite to those of lithium ions, as well as the current transmission direction, this leads to a large concentration polarization and high resistance [49]. What is more, low t_Li+_ combined with the low conductivity results in a low energy density, as well as cycling instability. By calculating from Figure 3c and Figure 4b, the t_Li+_ of PEG_H/L2000_ and PEG_H/L4000_ are 0.60 and 0.88, higher than most PEO-based electrolytes (Table S1). The results further confirm that the Lewis acid groups (–COO^−^ and –C=O) can enhance the anion capture ability of polymer electrolytes, which release a number of lithium ions and reinforce the transferability of lithium ions in PEG_H/L_.

Cyclic voltammetry (CV) tests in Figure 4d were performed in the Li|PEG_H/L2000_|LiFePO_4_ cell and Li|PEG_H/L4000_|LiFePO_4_ cell. The oxidized peaks of PEG_H/L2000_ and PEG_H/L4000_ are 3.9 V and 3.75 V, and the reduced peaks are 2.7 V and 3.1 V, respectively. Compared with the commercial cells with liquid electrolytes and LiFePO_4_ as the cathodes, the cells with PEG_H/L2000_ as the electrolytes show differences in the peak position, which may be due to the spontaneous side-reactions between PEG_H/L2000_ and lithium ions, while the cells assembled with PEG_H/L4000_ show no peak position difference compared with the peaks of the commercial cell [50].

The electrochemical windows of polymer solid electrolytes are mainly determined by the number of anions. The freer the anions, the easier electrolytes are oxidized under high potential, which limits the electrochemical windows of electrolytes generally under 4.0 V (vs. Li^+^/Li) [51,52], and further generates poor interfaces between electrolytes and electrodes, poor cycling stability, and low coulombic efficiency in the cycling process [53]. Electrochemical stabilization windows in Figure 4e were tested by linear sweep voltammetry (LSV). The LSV tests were performed on PEG_H/L2000_ and PEG_H/L4000_ with the stabilization window results of about 5 V (vs. Li^+^/Li) and 5.18 V (vs. Li^+^/Li), respectively, which is wider than most of the polymer electrolytes in Table S1 [21,54,55,56,57,58,59]. In comparison, the LSV tests were also conducted on PEG_LiTFSI_ under the same conditions, which shows the stabilization window is only about 3.8 V. The outcome indicates that the electron-deficient Lewis acid groups can capture a number of anions through Lewis acid–base reactions, which is important to reduce free anions in the electrolytes and broaden the electrochemical windows.

Figure S6 displays the electrochemical impedance spectroscopy (EIS) tests of PEG_LiTFSI_ and PEG_H/L_ at room temperature. It is worth noting that the impedance of PEG_LiTFSI_ shows a large capacitive reactance arc. By contrast, the Warburg diffusion impedance of PEG_H/L_ is extremely small and not visually distinct in the spectrum, which indicates that PEG_H/L_ has good interface contact to the electrodes. Moreover, the low impedance of PEG_H/L_ certifies that the compatibility problems of blending rigid and flexible segments are solved by the molecular-level reactions between HDI and PEG, in which the whole electrolyte system can be considered as a discontinuous hard phase distributed in a soft phase [40].

Conductivity comprises ionic conductivity and electronic conductivity in the solid electrolytes, and the electronic conductivity of the solid polymer electrolyte membranes was determined to ensure their function as effective ionic conductors and electronic insulators, which is crucial for preventing internal short circuits in a battery cell. The electronic conductivities of PEG_H/L2000_ and PEG_H/L4000_ in Figure 4f are 2.62 × 10^−10^ S·cm^−1^ and 8.74 × 10^−11^ S·cm^−1^ at room temperature, according to Equation (2). These exceptionally low electronic conductivity values (on the order of 10^−10^ to 10^−11^ S cm^−1^) confirm that the synthesized PEG_H/L_ membranes are predominantly ionic conductors with negligible electronic leakage, making them suitable for application in all-solid-state batteries. Therefore, it is convincing that the conductivity of PEG_H/L_ has been improved through the ionic conductivity rather than the electron conductivity because of the high lithium-ion transference numbers. As a result, the whole conductivities of PEG_LiTFSI_, PEG_H/L2000,_ and PEG_H/L4000_ are calculated to be 6.69 × 10^−5^ S·cm^−1^, 5.87 × 10^−4^ S·cm^−1,^ and 9.44 × 10^−4^ S·cm^−1^, respectively, in which the PEG_H/L4000_ shows higher conductivity than most of the PEO-based electrolytes (Table S1).

**Figure 5:**
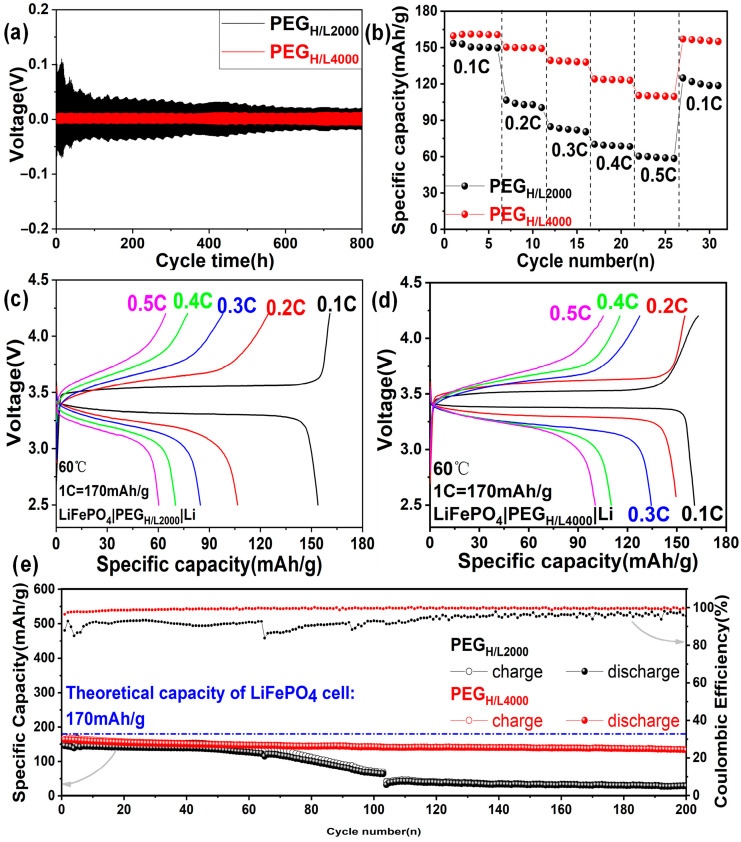
(**a**) Polarization tests at 60 °C by Li|PEG_H/L2000_|Li cells and Li|PEG_H/L4000_|Li cells; (**b**) rate capability at 60 °C of LiFePO_4_|PEG_H/L2000_|Li cells and LiFePO_4_|PEG_H/L4000_|Li cells ranged from 0.1 C to 0.5 C; charge and discharge profiles of (**c**) LiFePO_4_|PEG_H/L2000_|Li cells and (**d**) LiFePO_4_|PEG_H/L4000_|Li cells at different rates at 60 °C; (**e**) cycling performances and coulombic efficiencies at 0.1 C of the LiFePO_4_|PEG_H/L2000_|Li cells and LiFePO_4_|PEG_H/L4000_|Li cells ranged from 2.5 V to 4.2 V at 60 °C.

Galvanostatic charge–discharge tests have been compared between PEG_H/L2000_ and PEG_H/L4000_, since PEG_LiTFSI_ cannot successfully conduct the cycling test with an extremely high impedance (Figure S6). The polarization tests at 60 °C by lithium plating and stripping electrochemical cycling are displayed in Figure 5a, which were performed at a current density of 0.1 mA·cm^−2^ on the symmetric Li|PEG_H/L2000_|Li and Li|PEG_H/L4000_|Li cells. The Li|PEG_H/L2000_|Li cells show an overpotential for the plating and stripping process of about 0.1 V at the beginning and then gradually decrease to 0.03 V after cycling for 450 h and finally remain stable. It can be explained that the interface activation of electrolytes needs a long time, referring to the formation and decomposition of the solid electrolyte interphase (SEI) between PEG_H/L2000_ and electrode materials. That is to say, until cycling for more than 450 h, the SEI layer finally stabilized [50]. By contrast, the overpotential of symmetric cells with PEG_H/L4000_ is about 0.02 V initially and maintains this low and stable voltage polarization for more than 800 h, which indicates the target electrolyte PEG_H/L4000_ has obtained a highly stable lithium plating and stripping cycling reversibility.

Figure 5b exhibits the rate performances of the LiFePO_4_|PEG_H/L2000_|Li cell and LiFePO_4_|PEG_H/L4000_|Li cell at 60 °C. The specific capacities delivered by LiFePO_4_|PEG_H/L2000_|Li cells are 153.4 mAh·g^−1^, 106.6 mAh·g^−1^, 84.7 mAh·g^−1^, 70.1 mAh·g^−1^, and 60.3 mAh·g^−1^ at various rates of 0.1 C, 0.2 C, 0.3 C, 0.4 C, and 0.5 C. Better than that of the cells with PEG_H/L2000_, LiFePO_4_|PEG_H/L4000_|Li cell delivers higher specific capacities, which are reached at 160.8 mAh·g^−1^, 150.3 mAh·g^−1^, 139.4 mAh·g^−1^, 124.0 mAh·g^−1^, and 110.4 mAh·g^−1^ at the rate range from 0.1 C to 0.5 C, respectively. After completing larger current charging and discharging cycles, the capacity of the LiFePO_4_|PEG_H/L4000_|Li cell is basically restored to the original level when returning to the small current, with a high-capacity retention ratio of 98.4%. These results indicate that PEG-based disordered electrolytes can maintain the active materials’ stability during the rate performances ranging from 0.1 C to 0.5 C [60].

Figure 5c,d is the charge and discharge profiles of the LiFePO_4_|PEG_H/L2000_|Li cell and LiFePO_4_|PEG_H/L4000_|Li cell at different rates at 60 °C. It can be seen from Figure 5c that the LiFePO_4_|PEG_H/L2000_|Li cell has no obvious potential plateaus except for the rate of 0.1 C, indicating that side reactions may occur during the charge and discharge process, which is reflected by the comparatively low-capacity retention ratio of 81.4% when returning to the small current of 0.1 C. By contrast, the LiFePO_4_|PEG_H/L4000_|Li cell in Figure 5d has clear potential plateaus during the charge and discharge process, testifying that a higher reversible capacity has been obtained and side reactions are barely occurred during the charge and discharge process, which can also be demonstrated by its high-capacity retention ratio of 98.4% when returning to the small current of 0.1 C.

Cycling performances in Figure 5e at the rate of 0.1 C at 60 °C show the initial specific capacities of 145.8 mAh·g^−1^ and 163.1 mAh·g^−1^ for the LiFePO_4_|PEG_H/L2000_|Li and LiFePO_4_|PEG_H/L4000_|Li cells. It should be noted that their initial capacities show a small difference compared to the huge difference in lithium-ion transference numbers, because the cycling performances were conducted at a low current rate of 0.1 C. The initial coulombic efficiencies (ICEs) of the LiFePO_4_|PEG_H/L2000_|Li and LiFePO_4_|PEG_H/L4000_|Li cells are up to 87.9% and 96.5%, which can be comparable to PEG-based cells reported in the literature [32,61,62]. The cycling coulombic efficiencies (CCEs) of the two cells with PEG_H/L2000_ and PEG_H/L4000_ as electrolytes are 95.6% and 99.6% with capacity retentions of 20% (LiFePO_4_|PEG_H/L2000_|Li) and 82% (LiFePO_4_|PEG_H/L4000_|Li) after cycling for 200 cycles (Figure 5e), respectively, which demonstrates the electrodes maintain excellent interface structures with PEG_H/L_ after forming solid electrolyte interphase (SEI) in the first few cycles.

The better performances in cycling stability and rate capability of PEG_H/L4000_ than those of PEG_H/L2000_ can be attributed to the higher amorphous proportion of polymer electrolytes with higher PEG molecular weights at room temperature, which is beneficial to improve the conductivity and the transferability of lithium ions. The PEG_H/L4000_ with longer PEG chains (higher MW) between HDI cross-links provides a more continuous pathway for ion transport via segmental motion, leading to higher ionic conductivity (9.44 × 10^−4^ vs. 5.87 × 10^−4^ S·cm^−1^). The lower density of chain-ends and cross-links also contributes to a lower glass transition temperature (Tg), as seen in the DSC data, further enhancing mobility. In addition, it is reported that PEG or PEO with a relatively higher molecular weight will have better film formability and thermostability, which can provide enhanced mechanical performance and durability for solid-state electrolytes [30]. In contrast, the PEG_H/L2000_ with shorter chains (lower MW) results in a higher cross-linking density, which restricts chain mobility and explains its lower conductivity and higher Tg. This denser network, however, might contribute to its slightly better mechanical strength compared to a pure PEG_LiTFSI_, though it is still inferior to the PEG_H/L4000_ system.

## 4. Conclusions

In this work, from the perspective of building the disordered structures of PEG molecules, the brand-new polymer electrolyte PEG_H/L4000_ is synthesized with Lewis acid groups, as well as alternating rigid and flexible segments. The most important innovation is the introduction of the hard segment HDI blocks, which not only bridge links between the PEG molecules but also provide sites for generating Lewis acid groups and indirectly grafting lithium ions. The DFT calculations of binding energies between Lewis acid groups and lithium anions for the PEG_H/L4000_ certify that the electrolytes possess a strong anion-capture ability to release more free lithium ions. With the disordered structures, PEG_H/L4000_ exhibits superior electrochemical performances with lithium-ion transference numbers of 0.88, electrochemical windows up to 5.18 V (vs. Li^+^/Li), and a high conductivity of 9.44 × 10^−4^ S·cm^−1^. The polarization tests of symmetric cells with PEG_H/L4000_ show a low and stable voltage polarization at 0.02 V for more than 800 h. The rate performances of LiFePO_4_|PEG_H/L4000_|Li cells show high specific capacities at various rates, with the capacity retention ratio up to 98.4%. What is more, cycling performance tests at 60 °C for the cells with PEG_H/L4000_ as electrolytes exhibit high specific discharge capacities of 163.1 mAh·g^−1^ at the first cycle with an initial coulombic efficiency up to 96.5%, as well as a high 200th cycling coulombic efficiency up to 99.6%. The target PEG_H/L4000_ products with excellent performances provide a promising approach to the manufacture of all-solid-state lithium batteries with high safety as well as long life.

## Data Availability

Data is unavailable due to privacy or ethical restrictions.

## Supplementary Materials

The following supporting information can be downloaded at: https://www.mdpi.com/article/10.3390/mi16101123/s1, Figure S1: Synthesis procedures of (a) PEG_H/L_ and (b) PEG_LiTFSI_; (c) Schematic illustration of rigid and flexible chains alternated PEG_H/L_; Figure S2: FTIR spectra of DMPA and the obtained LiDMPA; Figure S3: ^1^H-NMR spectra of (a) DMPA, (b) LiDMPA and (c) HDI; Figure S4: FESEM images of PEG_H/L_; Figure S5: XRD patterns of PEG_LiTFSI_ and PEG_H/L_; Figure S6: Impedance of electrolytes at room temperature; Table S1: Comparison of some electrochemical performances of polymer electrolytes [21,54,55,56,57,58,59].

## References

[1] Zhang Y.M., Wu C.S., Yan Y.E., Li D.-S., Yang H.Y. (2025). In Situ Electrochemical Oxidation for High-Energy-Density Aqueous Batteries: Mechanisms, Materials, and Prospects. Adv. Mater..

[2] Song Z., Ma Y., Cheng X., Zhu Z., Zhong Y., He J., Wang T., Xu D., Zhang Q., Ozoemena K.I. (2025). Development of advanced anodes for solid-state lithium batteries. Mater. Today.

[3] Zhang Y.M., Zeng L.X., Ding Z.H., Wu W., Deng L.B., Yao L. (2023). Stable electrode/electrolyte interfaces regulated by dual-salt and localized high-concentration strategies for high-voltage lithium metal batteries. Chem. Commun..

[4] Yu Y., Ling C., Yang J., Zhu A., Naren T., Chen Y., Chen Y., Wei W., Ji X., Li C. (2024). Self-Healing fluorinated polymer deep eutectic electrolytes for stable lithium metal batteries. Chem. Eng. J..

[5] Wang Z., He Z., Wang Z., Long K., Yang J., Huang S., Wu Z., Mei L., Chen L. (2025). Engineering the solid electrolyte interphase for enhancing high-rate cycling and temperature adaptability of lithium-ion batteries. Chem. Sci..

[6] Fenton D.E., Parker J.M., Wright P.V. (1973). Complexes of alkali metal ions with poly(ethylene oxide). Polymer.

[7] Berthier C., Gorecki W., Minier M., Armand M.B., Chabagno J.M., Rigaud P. (1983). Polymer solid electrolytes—An overview. Solid State Ion..

[8] Huang B., Li Z.H., Zhu Y.M., Che Y., Wang C.A. (2022). Tailored lithium metal/polymer electrolyte interface with LiTa_2_PO_8_ fillers in PEO-based composite electrolyte. Rare Met..

[9] Qiu J., Liu X., Chen R., Li Q., Wang Y., Chen P., Gan L., Lee S.-J., Nordlund D., Liu Y. (2020). Enabling stable cycling of 4.2 V high-voltage all-solid-state batteries with PEO-based solid electrolyte. Adv. Funct. Mater..

[10] Zhang X.-Q., Chen X., Cheng X.-B., Li B.-Q., Shen X., Yan C., Huang J.-Q., Zhang Q. (2018). Highly Stable Lithium Metal Batteries Enabled by Regulating the Solvation of Lithium Ions in Nonaqueous Electrolytes. Angew. Chem. Int. Ed..

[11] Qu H., Zhang J., Du A., Chen B., Chai J., Xue N., Wang L., Qiao L., Wang C., Zang X. (2018). Multifunctional Sandwich-Structured Electrolyte for High-Performance Lithium–Sulfur Batteries. Adv. Sci..

[12] Wang C., Wang T., Wang L., Hu Z., Cui Z., Li J., Dong S., Zhou X., Cui G. (2019). Differentiated Lithium Salt Design for Multilayered PEO Electrolyte Enables a High-Voltage Solid-State Lithium Metal Battery. Adv. Sci..

[13] Wang Y., Wang G., He P., Hu J., Jiang J., Fan L.-Z. (2020). Sandwich structured NASICON-type electrolyte matched with sulfurized polyacrylonitrile cathode for high performance solid-state lithium-sulfur batteries. Chem. Eng. J..

[14] Lin D., Liu Y., Liang Z., Lee H.-W., Sun J., Wang H., Yan K., Xie J., Cui Y. (2016). Layered reduced graphene oxide with nanoscale interlayer gaps as a stable host for lithium metal anodes. Nat. Nanotechnol..

[15] Liu S., Xia X., Zhong Y., Deng S., Yao Z., Zhang L., Cheng X.-B., Wang X., Zhang Q., Tu J. (2018). 3D TiC/C Core/Shell Nanowire Skeleton for Dendrite-Free and Long-Life Lithium Metal Anode. Adv. Energy Mater..

[16] Zhang R., Cheng X.-B., Zhao C.-Z., Peng H.-J., Shi J.-L., Huang J.-Q., Wang J., Wei F., Zhang Q. (2016). Conductive Nanostructured Scaffolds Render Low Local Current Density to Inhibit Lithium Dendrite Growth. Adv. Mater..

[17] Deng K., Han D., Ren S., Wang S., Xiao M., Meng Y. (2019). Single-ion conducting artificial solid electrolyte interphase layers for dendrite-free and highly stable lithium metal anodes. J. Mater. Chem. A.

[18] Duan H., Yin Y.-X., Shi Y., Wang P.-F., Zhang X.-D., Yang C.-P., Shi J.-L., Wen R., Guo Y.-G., Wan L.-J. (2018). Dendrite-Free Li-Metal Battery Enabled by a Thin Asymmetric Solid Electrolyte with Engineered Layers. J. Am. Chem. Soc..

[19] Han X., Xu M., Gu L.H., Lan C.F., Chen M.F., Lu J.J., Sheng B.F., Wang P., Chen S.Y., Chen J.Z. (2024). Monothetic and conductive network and mechanical stress releasing layer on micron-silicon anode enabling high-energy solid-state battery. Rare Met..

[20] Hooper R., Lyons L.J., Moline D.A., West R. (1999). A Highly Conductive Solid-State Polymer Electrolyte Based on a Double-Comb Polysiloxane Polymer with Oligo(ethylene oxide) Side Chains. Organometallics.

[21] Zhao Z., Zhang Y., Li S., Wang S., Li Y., Mi H., Sun L., Ren X., Zhang P. (2019). A lithium carboxylate grafted dendrite-free polymer electrolyte for an all-solid-state lithium-ion battery. J. Mater. Chem. A.

[22] Qiao J., Chen Y., Baker G.L. (1999). Polymer Electrolytes Based on Unsaturated Ethylene Oxide-Segmented Polymers. Chem. Mater..

[23] Takagishi T., Okuda S., Kuroki N., Kozuka H. (1985). Binding of cupric ion by crosslinked polyethylenimine. J. Polym. Sci. Polym. Chem. Ed..

[24] Wu J.C., Gao S.B., Li X.W., Zhou H.T., Gao H.Q., Hu J.L., Fan Z.H., Liu Y.J. (2024). Rigid-flexible coupling network solid polymer electrolytes for all-solid-state lithium metal batteries. J. Colloid Interface Sci..

[25] Hawker C.J., Chu F., Pomery P.J., Hill D.J.T. (1996). Hyperbranched Poly(ethylene glycol)s:  A New Class of Ion-Conducting Materials. Macromolecules.

[26] Zhang Z., Sherlock D., West R., Amine K., Lyons L.J. (2003). Cross-Linked Network Polymer Electrolytes Based on a Polysiloxane Backbone with Oligo(oxyethylene) Side Chains:  Synthesis and Conductivity. Macromolecules.

[27] Nishimoto A., Agehara K., Furuya N., Watanabe T., Watanabe M. (1999). High Ionic Conductivity of Polyether-Based Network Polymer Electrolytes with Hyperbranched Side Chains. Macromolecules.

[28] Khurana R., Schaefer J.L., Archer L.A., Coates G.W. (2014). Suppression of Lithium Dendrite Growth Using Cross-Linked Polyethylene/Poly(ethylene oxide) Electrolytes: A New Approach for Practical Lithium-Metal Polymer Batteries. J. Am. Chem. Soc..

[29] Kurono R., Mehta M.A., Inoue T., Fujinami T. (2001). Preparation and characterization of lithium ion conducting borosiloxane polymer electrolytes. Electrochimica Acta.

[30] Li Q., Cao D.X., Naik M.T., Pu Y.Q., Sun X., Luan P.C., Ragauskas A.J., Ji T.T., Zhao Y.Y., Chen F.Q. (2022). Molecular Engineering of Biorefining Lignin Waste for Solid-State Electrolyte. ACS Sustain. Chem. Eng..

[31] Li Y.X., Yang J., Zhang X.Z., Cui X.M., Pan Q.M. (2023). Enhancing Li ion conduction through polyethylene glycol brushes towards long-life solid-state lithium metal batteries. J. Mater. Chem. A.

[32] Zhou S., Wang X., Xu Z., Guan T., Mo D., Deng K. (2024). Rapid self-healing, highly conductive and near-single-ion conducting gel polymer electrolytes based on dynamic boronic ester bonds for high-safety lithium metal batteries. J. Energy Storage.

[33] Kamiyama Y., Shibata M., Kanzaki R., Fujii K. (2020). Lithium-ion coordination-induced conformational change of PEG chains in ionic-liquid-based electrolytes. Phys. Chem. Chem. Phys..

[34] Delley B. (2000). From Molecules to Solids with the DMol3 Approach. J. Chem. Phys..

[35] Delley B. (2002). Hardness conserving semilocal pseudopotentials. Phys. Rev. B.

[36] Perdew J.P., Burke K., Ernzerhof M. (1996). Generalized Gradient Approximation Made Simple. Phys. Rev. Lett..

[37] Grimme S. (2006). Semiempirical GGA-type density functional constructed with a long-range dispersion correction. J. Comput. Chem..

[38] Huang W., Zhao G., Zhang B., Li T., Zhang H., Wang J., Zhao X., Hu X., Xu Y. (2025). Recent Advances for Cation-Anion Aggregates in Solid Polymer Electrolytes: Mechanism, Strategies, and Applications. Small Methods.

[39] Yu F., Wang Y., Liu J., Zhang Y., Yang Z., Shen H., Li J., Zeng G., Zhang K., Cui D. (2025). High-Entropy Solid-State Electrolytes for Rechargeable Batteries: Mechanism, Structural Designs, Characterizations, and Applications. Small.

[40] Nederstedt H., Jannasch P. (2019). Single-ion conducting polymer electrolytes with alternating ionic mesogen-like moieties interconnected by poly(ethylene oxide) segments. Polymer.

[41] Chatt J., Duncanson L.A. (1953). Olefin co-ordination compounds. Part III. Infra-red spectra and structure: Attempted preparation of acetylene complexes. J. Chem. Soc. (Resumed).

[42] Bistoni G., Rampino S., Scafuri N., Ciancaleoni G., Zuccaccia D., Belpassi L., Tarantelli F. (2016). How π back-donation quantitatively controls the CO stretching response in classical and non-classical metal carbonyl complexes. Chem. Sci..

[43] Gillespie R.J. (1970). The electron-pair repulsion model for molecular geometry. J. Chem. Educ..

[44] Porcarelli L., Manojkumar K., Sardon H., Llorente O., Shaplov A.S., Vijayakrishna K., Gerbaldi C., Mecerreyes D. (2017). Single Ion Conducting Polymer Electrolytes Based on Versatile Polyurethanes. Electrochim. Acta.

[45] Fulmer G.R., Miller A.J.M., Sherden N.H., Gottlieb H.E., Nudelman A., Stoltz B.M., Bercaw J.E., Goldberg K.I. (2010). NMR Chemical Shifts of Trace Impurities: Common Laboratory Solvents, Organics, and Gases in Deuterated Solvents Relevant to the Organometallic Chemist. Organometallics.

[46] Mukai T., Yoshio M., Kato T., Ohno H. (2004). Effect of Methyl Groups onto Imidazolium Cation Ring on Liquid Crystallinity and Ionic Conductivity of Amphiphilic Ionic Liquids. Chem. Lett..

[47] Das D., Chandrasekaran A., Venkatram S., Ramprasad R. (2018). Effect of Crystallinity on Li Adsorption in Polyethylene Oxide. Chem. Mater..

[48] Qiu F., Li X., Deng H., Wang D., Mu X., He P., Zhou H. (2019). A Concentrated Ternary-Salts Electrolyte for High Reversible Li Metal Battery with Slight Excess Li. Adv. Energy Mater..

[49] Mindemark J., Lacey M.J., Bowden T., Brandell D. (2018). Beyond PEO—Alternative host materials for Li+-conducting solid polymer electrolytes. Prog. Polym. Sci..

[50] Li H., Liu W., Yang X., Xiao J., Li Y., Sun L., Ren X., Zhang P., Mi H. (2021). Fluoroethylene carbonate-Li-ion enabling composite solid-state electrolyte and lithium metal interface self-healing for dendrite-free lithium deposition. Chem. Eng. J..

[51] Xiao L., Zeng Z., Liu X., Fang Y., Jiang X., Shao Y., Zhuang L., Ai X., Yang H., Cao Y. (2019). Stable Li Metal Anode with “Ion–Solvent-Coordinated” Nonflammable Electrolyte for Safe Li Metal Batteries. ACS Energy Lett..

[52] Basile A., Bhatt A.I., O’MullanS1e A.P. (2016). Stabilizing lithium metal using ionic liquids for long-lived batteries. Nat. Commun..

[53] Chang Z., Qiao Y., Yang H., Deng H., Zhu X., He P., Zhou H. (2020). Beyond the concentrated electrolyte: Further depleting solvent molecules within a Li+ solvation sheath to stabilize high-energy-density lithium metal batteries. Energy Environ. Sci..

[54] Shim J., Kim D.G., Kim H.J., Lee J.H., Lee J.C. (2015). Polymer Composite Electrolytes Having Core–Shell Silica Fillers with Anion-Trapping Boron Moiety in the Shell Layer for All-Solid-State Lithium-Ion Batteries ACS Appl. Mater. Interfaces..

[55] Zhang J., Ma C., Hou H., Li X., Chen L., Ivey D.G., Wei W. (2018). A star-shaped solid composite electrolyte containing multifunctional moieties with enhanced electrochemical properties for all solid-state lithium batteries. J. Membr. Sci..

[56] Chen H., Adekoya D., Hencz L., Ma J., Chen S., Yan C., Zhao H.J., Cui G.L., Zhang S.Q. (2020). Stable Seamless Interfaces and Rapid Ionic Conductivity of Ca-CeO2/LiTFSI/PEO Composite Electrolyte for High-Rate and High-Voltage All-Solid-State Battery. Adv. Energy Mater..

[57] Li Z., Li A., Zhang H., Lin R., Jin T., Cheng Q., Xiao X.H., Lee W.-K., Ge M.Y., Zhang H.J. (2020). Interfacial engineering for stabilizing polymer electrolytes with 4V cathodes in lithium metal batteries at elevated temperature. Nano Energy.

[58] Liu C., Wang J., Kou W., Yang Z., Zhai P., Liu Y., Wu W., Wang J. (2021). A flexible, ion-conducting solid electrolyte with vertically bicontinuous transfer channels toward high performance all-solid-state lithium batteries. Chem. Eng. J..

[59] Cheng S.H.S., Liu C., Zhu F., Zhao L., Fan R., Chung C.Y., Tang J.N., Zeng X.R., He Y.B. (2021). (Oxalato)borate: The key ingredient for polyethylene oxide based composite electrolyte to achieve ultra-stable performance of high voltage solid-state LiNi_0.8_Co_0.1_Mn_0.1_O_2_/lithium metal battery. Nano Energy.

[60] Zhu J., Zhang Z., Zhao S., Westover A.S., Belharouak I., Cao P.-F. (2021). Single-ion conducting polymer electrolytes for solid-state lithium–metal batteries: Design, performance, and challenges. Adv. Energy Mater..

[61] Rong Z., Sun Y., Yang M., Cheng F., Zhang W., Chen J. (2023). How the PEG terminals affect the electrochemical properties of polymer electrolytes in lithium metal batteries. Energy Storage Mater..

[62] Zhu X., Fang Z., Deng Q., Zhou Y., Fu X., Wu L., Yan W., Yang Y. (2022). Poly(ionic liquid) @PEGMA Block Polymer Initiated Microphase Separation Architecture in Poly (ethylene oxide)-Based Solid-State Polymer Electrolyte for Flexible and Self- Healing Lithium Batteries. ACS Sustain. Chem. Eng..

