# Supplementary material for: Polyethylene Glycol-Based Solid Polymer Electrolyte with Disordered Structure Design for All-Solid-State Lithium-Ion Batteries
Source: Micromachines (Basel). 2025 Sep 30;16(10):1123. doi: 10.3390/mi16101123 (PMC12566210; doi:10.3390/mi16101123)
Supplement: Supplementary file 1 [file micromachines-16-01123-s001.zip › micromachines-3878825-supplementary.pdf]

Supporting Information

Polyethylene Glycol-Based Solid Polymer Electrolyte with Disordered Structure Design for All-Solid-State Lithium-Ion Batteries

Wanlin Wu [1,†], Yingmeng Zhang [2,*,†], Zhongke Zhao [1], Yihan Lin [1], Yongliang Li [1], Xiangzhong Ren [1], Peixin Zhang [1] and Lingna Sun [1,*]

[1] College of Chemistry and Environmental Engineering, Shenzhen University, Shenzhen 518060, China
[2] Yangtze Delta Region Institute (Huzhou), University of Electronic Science and Technology of China, Huzhou 313000, China
* Correspondence: sunln@szu.edu.cn (L.S.); ymzhang@csj.uestc.edu.cn (Y.Z.); Tel.: +86-18005720116 (Y.Z.)
† These authors contributed equally to this work.

Figures

Chemical equations and models of $PEG_{H/L}$ and PEG_{LiTFSI}

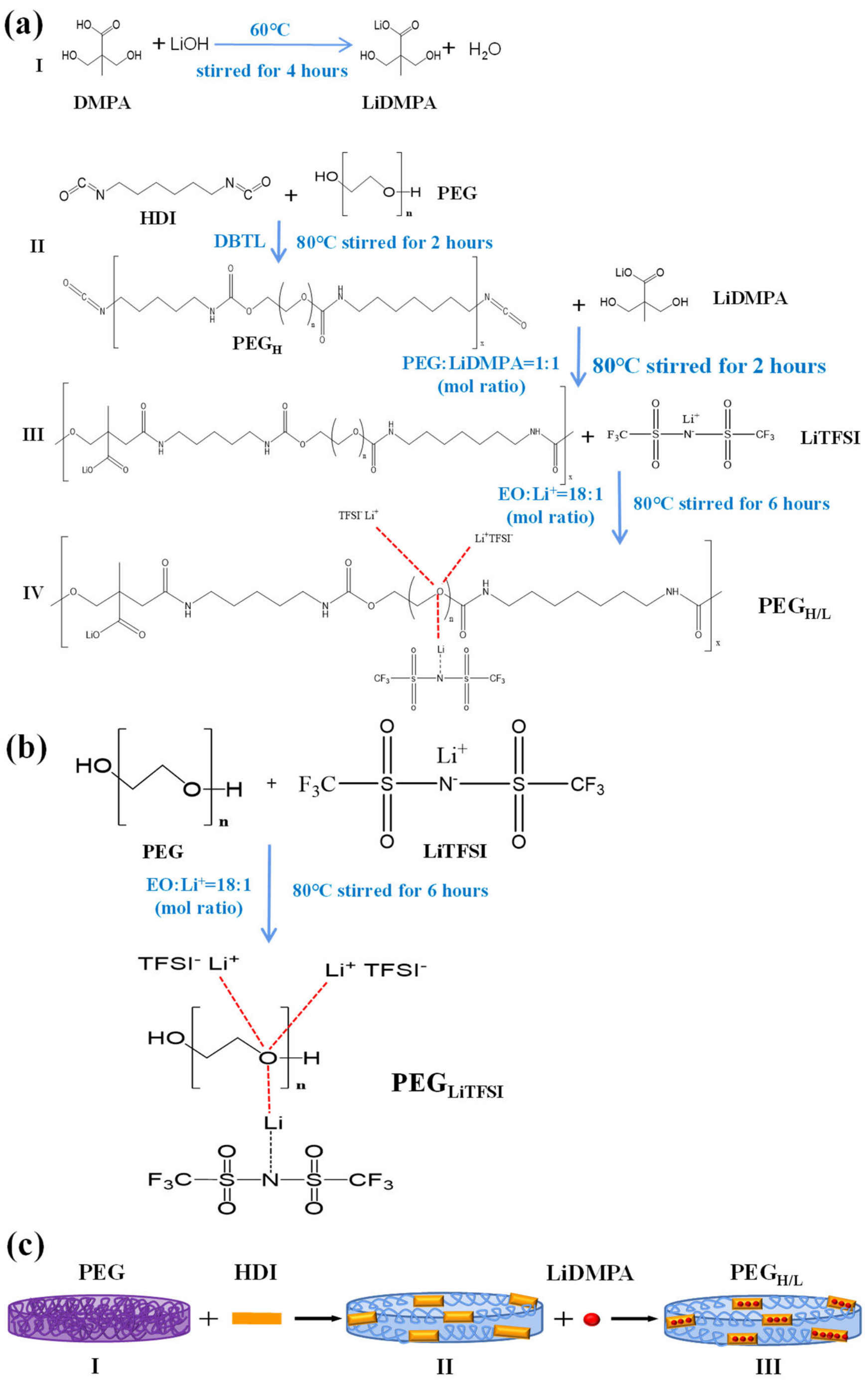

Figure S1. Synthesis procedures of (a) $PEG_{H/L}$ and (b) PEG_{LiTFSI}; (c) Schematic illustration of rigid and flexible chains alternated $PEG_{H/L}$.

FTIR spectra of precursor

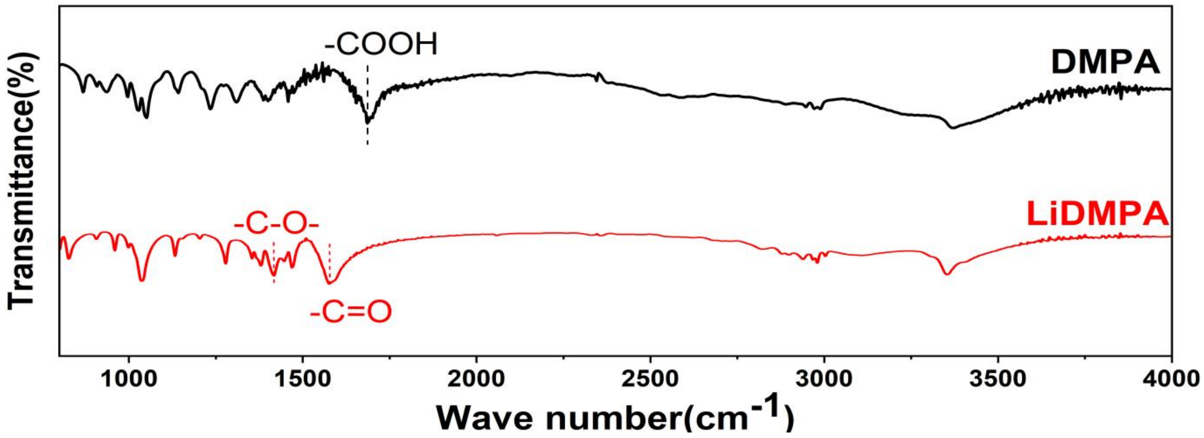

Figure S2. FTIR spectra of DMPA and the obtained LiDMPA.

NMR spectra of the reactant materials

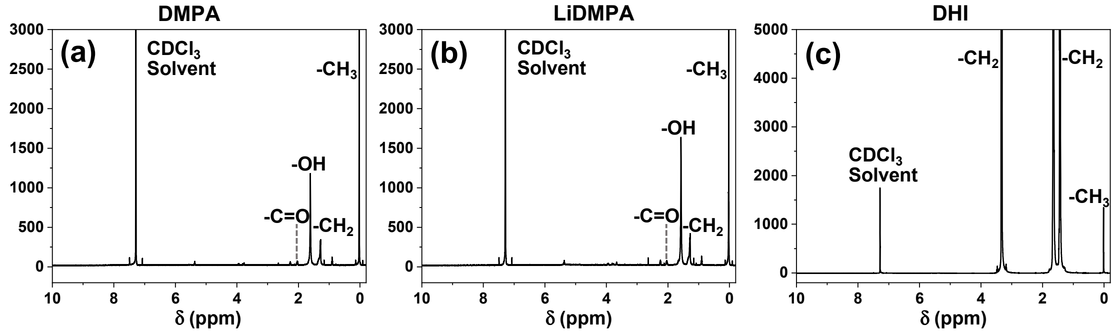

Figure S3. ^{1}H-NMR spectra of (a) DMPA, (b) LiDMPA and (c) HDI.

FESEM images of $PEG_{H/L}$

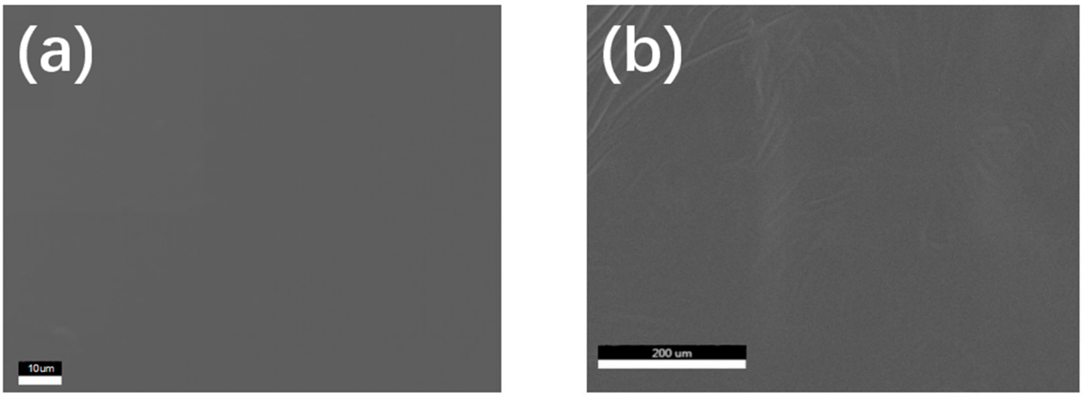

Figure S4. FESEM images of $PEG_{H/L}$.

XRD patterns

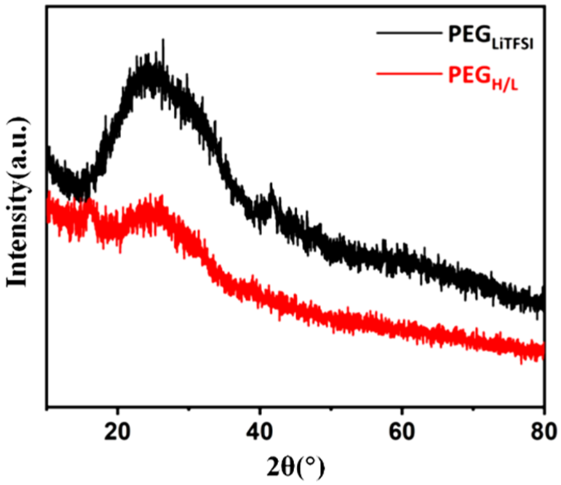

Figure S5. XRD patterns of PEG_{LiTFSI} and $PEG_{H/L}$

Impedance of electrolytes

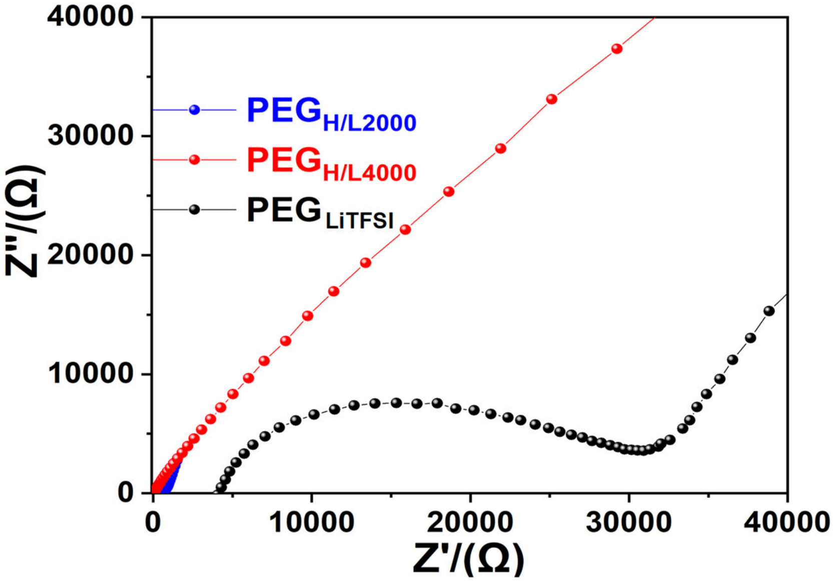

Figure S6. Impedance of electrolytes at room temperature.

Comparison of electrochemical performances of polymer electrolytes

Table S1. Comparison of some electrochemical performances of polymer electrolytes

Electrolyte composition	Ionic conductivity	Linear sweep voltammetry (LSV, vs.Li^+/Li)	t_{Li+}	Specific capacity/CCEs/C-rate	reference
$PEG_{H/L4000}$	9.44×10^{-4}	5.18 V	0.88	163.1 $mAh\cdot g^{-1}$, 82% (200th, 60°C), 0.1C	This work
$PEG_{H/L2000}$	5.87×10^{-4}	5.0 V	0.60	145.8 $mAh\cdot g^{-1}$, 20% (200th, 60°C), 0.1C	This work
PEG_{LiTFSI}	6.69×10^{-5}	3.8 V	0.06	/	This work
PEG2000/MDI/LiDMPA/LiTFSI	9.60×10^{-5}	5.0 V	0.72	159 $mAh\cdot g^{-1}$, 84% (100th, 60°C), 0.2C	5
PEG475/POSS/$LiClO_4$	1.60×10^{-4}	4.3 V	0.67	291 $mAh\cdot g^{-1}$, 80% (50th, 60°C), 0.1C	6

B-PEGMA/PEGMEM/OV-POSS/ LiTFSA	3.44×10^{-4}	5.8 V	0.58	131 mAh·g^{-1}, 91.6% (100th, 25°C), 0.5C	7
PEO/Ca-CeO_2/LiTFSI	1.30×10^{-4} (60°C)	4.2 V	0.45	121 mAh·g^{-1}, 35% (200th, 60°C), 1C	8
PEO/PEGDME/LiTFSI/LiBOB	2.30×10^{-4}	4.4 V		154.7 mAh·g^{-1}, 81.9% (150th, 25°C), 1C	9
PEO/$Li_{0.33}La_{0.56}TiO_3$(LLTO)/LiTFSI	2.04×10^{-4}	4.7 V	0.59	155.6 mAh·g^{-1}, 97.2% (150th, 60°C), 1C	10
PEO/$LiNi_{0.8}Co_{0.1}Mn_{0.1}O_2$(NCM811)/EC/ $Li_{6.4}La_3Zr_{1.4}Ta_{0.6}O_{12}$(LLZTO)/$LiClO_4$	9.00×10^{-4}	4.7 V		152 mAh·g^{-1}, 95% (200th, 25 °C), 0.2C	11

Reference

1. B. Delley, *J Chem Phys*, 2000,**18**, 7756-7764.
2. B. Delley, *Phys Rev B*, 2002, **15**, 155125.
3. J. P. Perdew; K. Burke; M. Ernzerhof, *Phys Rev Lett*, 1996, **18**, 3865--3868.
4. S. Grimme, *Journal of computational chemistry*, 2006, **27**, 1787-99.
5. Z. K. Zhao, Y. M. Zhang, S. J. Li, S. H. Wang, Y. L. Li, H. W. Mi, L. N. Sun, X. Z. Ren, P. X. Zhang, *Journal of Materials Chemistry A*, 2019, **7** , 25818-25823.
6. J. Shim, D-G. Kim, H. J. Kim, J. H. Lee, J-C. Lee, *ACS Applied Materials & Interfaces*, 2015, **7** , 7690-7701.
7. J. F. Zhang, C. Ma, H. Hou, X. F. Li, L. B. Chen, D. G. Ivey, W. F. Wei, *Journal of Membrane Science*, 2018, **552**, 107-114.
8. H. Chen, D. Adekoya, L. Hencz, J. Ma, S. Chen, C. Yan, H. J. Zhao, G. L. Cui, S. Q. Zhang, *Advanced Energy Materials* 2020, **10**, 2000049.
9. Z. Y. Li, A. J. Li, H. R. Zhang, R. Q. Lin, T. W. Jin, Q. Cheng, X. H. Xiao, W-K. Lee, M. Y. Ge, H. J. Zhang, A. Zangiabadi, I. Waluyo, A. Hunt, H. Zhai, J. J. Borovilas, P. Y. Wang, X-Q. Yang, X. Y. Chuan, Y. Yang, *Nano Energy*, 2020, **72**, 104655.
10. C. Liu, J. X. Wang, W. J. Kou, Z. H. Yang, P. F. Zhai, Y. Liu, W. J. Wu, J. T. Wang, *Chemical Engineering Journal*, 2021, **404,** 126517.

11. S. H-S. Cheng, C. Liu, F. Y. Zhu, L. Zhao, R. Fan, C-Y. Chung, J. N. Tang, X. R. Zeng, Y-B. He, *Nano Energy*, 2021, **80**, 105562.
